# The American Journal of Insanity

**Published:** 1845-08

**Authors:** 


THE MEDICAL EXAMINER.

PHILADELPHIA, AUGUST, 1845.

### THE AMERICAN JOURNAL OF INSANITY.

We have received the first number of the *Second Volume* of this interesting periodical, from which we are glad to discover that the enterprise is likely to succeed—of which, on its first announcement, we confess we had some doubts. We had indeed no conception that a journal limited to that subject, could be rendered so interesting as we have found it to be. But in the able hands by which it is conducted, the general subject is made to embrace a great number and variety of topics, all having a direct relation, however, to the great purposes of the publication. The articles which have appeared thus far, have treated of matters well calculated to interest the general as well as the professional reader, and are written with great ability.

